# Introducing an All-mechanized Surgical Assistant for Use in Reconstructive Surgeries

**DOI:** 10.1097/GOX.0000000000002403

**Published:** 2019-09-30

**Authors:** Yoshihiro Sowa, Ryo Yamochi, Takuya Kodama, Daiki Morita, Toshiaki Numajiri

**Affiliations:** From the Departments of Plastic and Reconstructive Surgery, Graduate School of Medical Sciences, Kyoto Prefectural University of Medicine, Kyoto, Japan.

## Sir,

There is currently a critical lack of surgeons in many advanced countries, including Japan. A geographic shortage of plastic surgeons is also sometimes considered to be a social problem.^1,2^ The key to solving this issue depends on proper and thoughtful deployment of surgical resources and on how smoothly the medical education system can provide residents with appropriate educational opportunities. During surgeries, surgical assistants often do nothing but simple tasks such as keeping the surgical field clear and simply follow top-down orders throughout the operation. Performance of these activities is a waste of talent and time for an assistant who has similar or higher skills than the senior surgeon. Alternatively, if the assistant is at the training level, he or she rarely gets effective learning opportunities due to standing in a position from which it is not possible to see the surgical field clearly. This situation provides young surgeons with nothing but a very poor view and position from which to have new surgical ideas.

A totally automated operation could be one solution to these problems. Automated, unmanned hotels and retail services are becoming common. Machines and robots have already replaced people for helpful and essential assistance in daily life, and utilization of similar systems in the role of a surgical assistant is plausible. A good example is provided by the Octopus (MEDNOSBRO GmbH, Rudolfstetten, Switzerland), a device that serves as a versatile retractor system, and has 3 joints similar to those in a human upper limb, which allows precise all-direction maneuvers. In addition to this function, the system has flexible settings that allow a surgeon to place the machine in ideal positions to have a perfect view only by tightening or loosening a special screw attached to the device. A single surgeon can then complete an entire surgery using various tip parts of the retractor. Just like an assistant, the system plays a major role in the surgery, including facilitating a variety of flap elevations.

Our surgical team has introduced the Octopus device into many kinds of reconstructive surgery, although mainly breast reconstruction, and we have shown that many plastic and reconstructive surgeries can be performed without an assistant. The device enables a single surgeon to complete the surgery alone, even in emergency situations, and never disturbs the surgeon’s work, loses focus, or becomes tired. The device makes it possible to reduce labor costs and to place sufficient talent in suitable positions, which releases assistants from unfruitful positions and consequently allows them to contribute much more in another field. If assistants are inexperienced residents, they can freely observe a surgery with a clear view that may not differ from the surgeon’s point of view, which allows acquisition of an understanding of a detailed procedure in an efficient way.

There have been many beneficial results of device-assisted operations in various surgical fields,^3–5^ but relatively few in plastic and reconstructive surgery. However, this approach has great potential in this field, and we believe that this new surgical concept will spread quickly in the future.

**Fig. 1.**
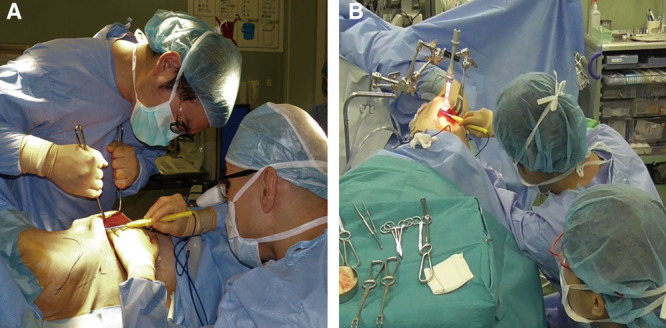
Surgical assistants standing in traditional positions, from which they often cannot see the surgical field clearly (A). With the use of an automated device, surgical assistants can stand in a more beneficial position with the same view of the surgical field as that of the surgeon (B).

**Fig. 2.**
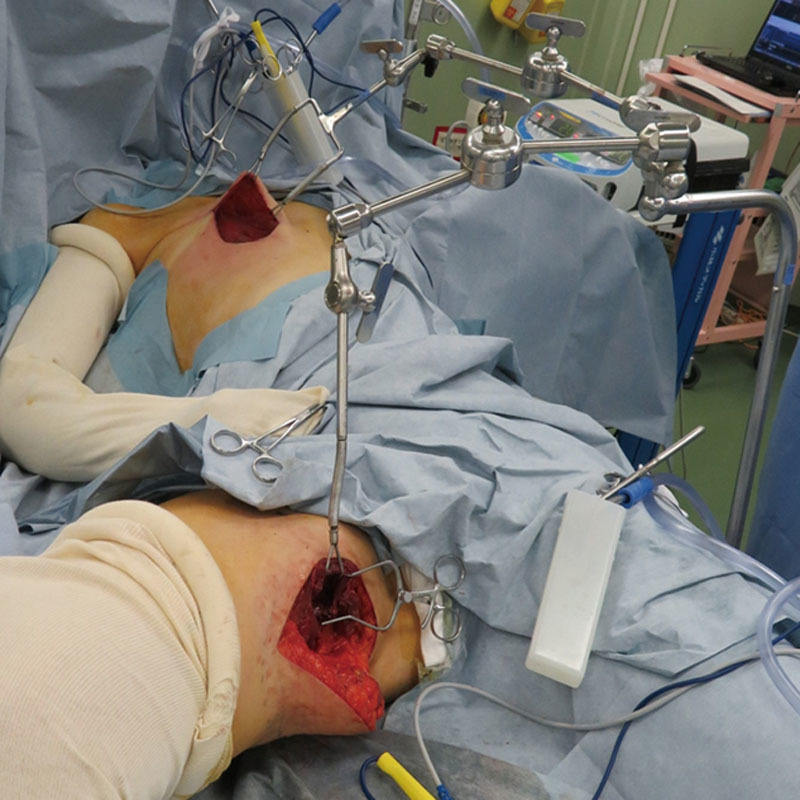
The versatile Octopus retractor system maintains the surgical field for profunda artery perforator flap harvest and for preparation of recipient vessels at the same time, as shown in this surgery for breast reconstruction.
